# Biomechanical Analysis of 3D Printed and Thermoformed EVA Custom‐Made Mouthguard: Experimental and Finite Element Analyses

**DOI:** 10.1111/edt.70044

**Published:** 2025-12-18

**Authors:** Airin Karelys Avendaño Rondon, Izabela Batista Cordeiro, Maribí Isomar Terán Lozada, Gustavo Mendonça, Priscilla Barbosa Ferreira Soares, Paulo Sergio Borella, Carlos José Soares

**Affiliations:** ^1^ Department of Operative Dentistry and Dental Materials, Dental School Federal University of Uberlândia, Uberlândia Uberlândia Minas Gerais Brazil; ^2^ Department of General Practice, School of Dentistry Virginia Commonwealth University Richmond Virginia USA; ^3^ Department of Periodontology and Implantology, School of Dentistry Federal University of Uberlândia Uberlândia Minas Gerais Brazil

**Keywords:** 3D printed polymer, dental trauma, ethylene‐vinyl acetate, finite element analysis, mechanical properties, mouthguard

## Abstract

**Background/Objective:**

3D printed materials have been evaluated for fabricating mouthguards; however, little is known about their biomechanical performance in preventing dental trauma. This study aimed to compare two 3D printed polymers and three ethylene vinyl acetate (EVA) sheets used for mouthguard fabrication regarding their mechanical properties and stress–strain behavior during impact, analyzed through finite element analysis (FEA).

**Materials and Methods:**

Two 3D printed polymers, KeyGuard (Keystone) and DimaPrint (Kulzer), and three EVA sheets, Bio‐art, Essence Dental, and Proform, were used to create the specimens following ISO 37‐II (*n* = 10). Shore A hardness was measured on the surface. The breaking force (*F*, N), elongation (EL, %), ultimate tensile strength (UTS, MPa), and elastic modulus (E, MPa) were measured using a universal testing machine (EMIC). Density (*ρ*, g/cm^3^) was determined using the test method following ASTM D792. Poisson's ratio was calculated by using axial tensile tests following ASTM D638. Scanning electron microscopy was used to examine the surfaces of EVAs and 3D printed polymers. A two‐dimensional finite element model of the maxillary structure and upper incisor was evaluated with and without a 4.0 mm mouthguard made from each material. An impact simulation was performed by striking the upper incisor with a rigid surface at 1 m/s (3.6 km/h). Strain and modified von Mises stress distributions were evaluated, and mouthguard displacement relative to the tooth was calculated.

**Results:**

One‐way ANOVA revealed significant differences among the tested mouthguards for all evaluated parameters (*p* < 0.01). Essence Dental showed the highest and DimaPrint the lowest values of *F* and UTS values (*p* < 0.01). Proform was the highest, and DimaPrint had the lowest EL values (*p* < 0.01). DimaPrint and KeyGuard had the highest, and Proform had the lowest E values (*p* < 0.01). KeyGuard, Essence Dental, and Bio‐art had higher Shore A values than Proform and DimaPrint (*p* < 0.01). The 3D printed polymer group had significantly higher Poisson's ratio values than EVAs. The density values were similar for all tested materials (*p* = 0.055). The absence of a mouthguard led to greater stress and strain on the impacted tooth. All mouthguards significantly reduced these values, showing comparable shock‐absorbing capacity. Displacement was generally lower in the buccal region and increased toward the palatal side.

**Conclusion:**

Although the 3D‐printed polymers exhibited mechanical properties different from EVA materials, all mouthguards demonstrated similar effectiveness for reducing stress and strain on the impacted tooth, indicating comparable protection against dental trauma.

## 1 Introduction

Dental trauma is one of the most frequent emergencies in oral healthcare, requiring immediate intervention due to its potential to cause functional and esthetic impairments [1, 2, 3]. Epidemiological data consistently indicate a high prevalence of orofacial trauma among athletes, with the maxillary central incisors being the most affected teeth [4, 5, 6]. Participation in sports is a well‐recognized determinant of these injuries, particularly in activities with frequent body contact or high‐energy impacts [5, 7]. Disciplines such as boxing, martial arts, and hockey are associated with a high probability of direct facial blows or impacts with equipment [7, 8, 9], whereas in sports like basketball or soccer, the lower intensity of collisions does not eliminate the risk since accidental contact between players, falls, or strikes from the ball remain common causes [8, 10]. The anterior position and projection of the maxillary central incisors explain why they are consistently the most vulnerable teeth in these scenarios [11, 12].

Mouthguards can prevent or minimize damage caused by different traumatic events, such as fractures involving teeth or restorations [13], injuries to adjacent soft and hard tissues [14] and damage to the mandibular condyle and articular disc [15, 16]. They can even prevent stress on the permanent germ in the case of deciduous teeth [17]. The performance of mouthguards depends on several factors, such as the type of material [5, 6], geometry, manufacturing process, and thickness [16, 18, 19].

Mouthguards have been produced by using EVA copolymer, polyvinyl acetate‐polyethylene, polyvinyl chloride, latex rubber, and polyurethane [20, 21, 22]. A commonly reported disadvantage of thermoforming is the marked decrease in material thickness that follows the molding stage, which diminishes its ability to provide adequate protection when compared with the original sheet [23]. Custom‐made mouthguards, using two EVA layers, have been used as an alternative to minimize this disadvantage [18, 22, 24]. Nonetheless, the conventional fabrication of EVA appliances demands extended production time [24, 25]. Among the ideal characteristics of the materials used for fabricating mouthguards, low water absorption, appropriate hardness, a low incidence of delamination [24], and excellent mechanical properties, such as hysteresis and elastic modulus, are required [26, 27]. Hysteresis refers to the disparity between the energy necessary to distort a material and the energy retained and subsequently released as the material reverts to its original form [27]. The elastic modulus quantifies a material's stiffness or rigidity. This mechanical property is essential for guaranteeing the precision and dependability of finite element analysis employed to evaluate the stress–strain behavior of mouthguards [13, 27, 28]. The mechanical properties of EVA have been thoroughly investigated, validating its efficacy in improving the performance of mouthguards [18, 19, 26].

The mouthguard's shape and thickness can be precisely controlled by using additive manufacturing, enhancing its customization and functionality [21]. 3D printing can also provide faster production, reducing the time required for mouthguard fabrication [29]. Recent advances in additive manufacturing have enabled the production of devices with adequate impact absorption and dimensional stability, highlighting the potential of this technology to replace conventional thermoforming techniques [30]. The 3D‐printed mouthguards fabricated from photopolymer resins [20, 25, 31], such as Dima Print Mouth Guard and KeyGuard, can achieve impact performance comparable to that of conventional EVA mouthguards [20]. However, most available data focus on the functional response of the printed mouthguards, while information regarding the material characterization and specific mechanical properties of these polymers remains limited. Therefore, the aim of this study was to assess the mechanical properties of two novel 3D printed polymers compared with EVAs used for custom‐made mouthguard fabrication, and to evaluate their biomechanical performance, as expressed by stress and strain values during impact simulation using finite element analysis. The null hypotheses were: 1. There are no significant differences in the mechanical properties between the 3D‐printed polymers and EVA materials used for custom‐made mouthguards, and 2. The mechanical properties of the new 3D printed polymers for producing mouthguards would not affect the stress (MPa) and strain (με) values of custom‐made mouthguards measured using finite element analysis.

## 2 Materials and Methods

**FIGURE 1:**
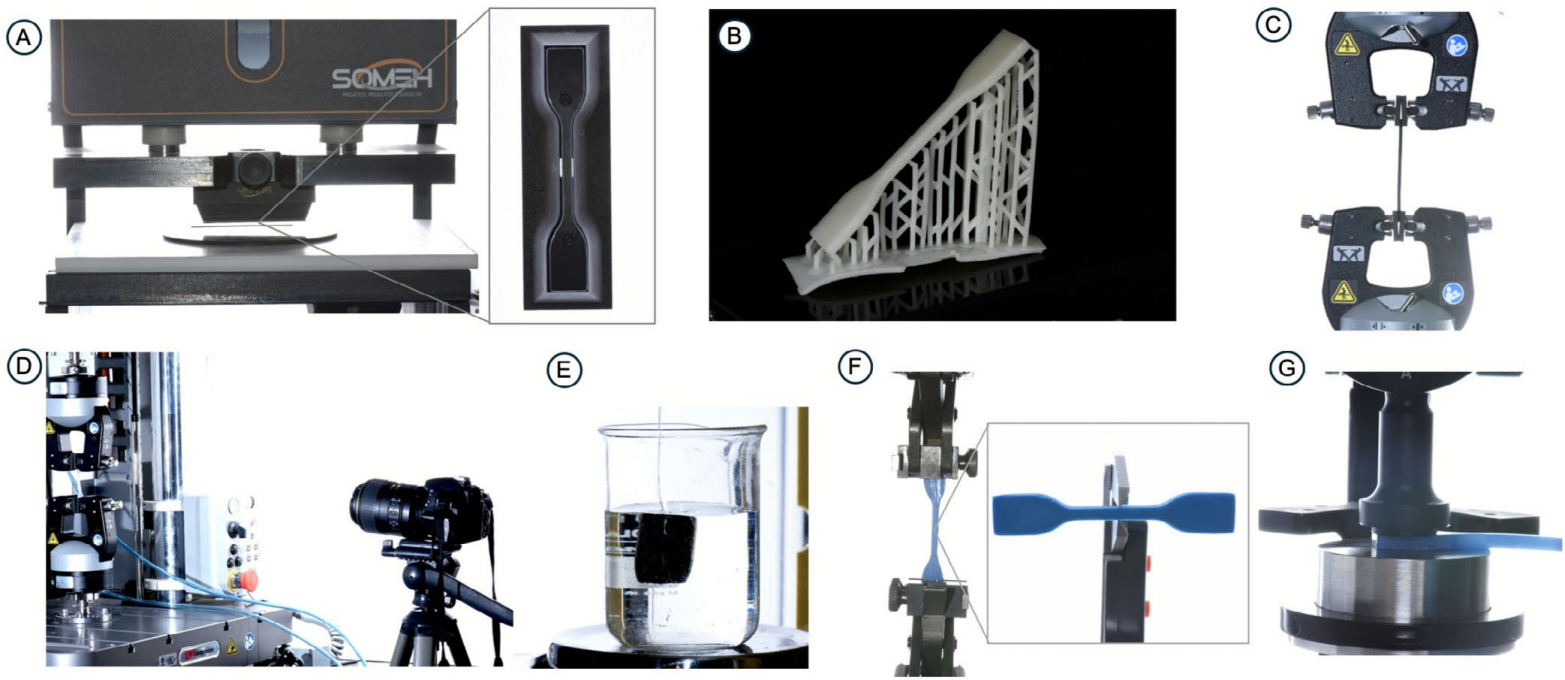
Specimen preparation and mechanical testing procedures. (A) Cutting dumbbell‐shaped specimens from EVA sheets using a metal mold. (B) Dumbbell‐shaped specimen fabricated by 3D printing. (C) Tensile test for calculating the elastic modulus. (D) Test to determine the Poisson's ratio of the tested materials. (E) Test to determine the density of the tested materials. (F) Dumbbell specimen positioned for axial tensile testing, with a magnified view of the sample. (G) Shore A hardness test, showing the durometer tip in contact with the surface of the specimen.

Three soft‐colored EVA sheets: Bio‐art (Bio‐art, São Carlos, Brazil), Essence Dental (Essence Dental, Ribeirão Preto, Brazil), and Proform (Sportsguard Laboratories, Kent, USA) (Table 1), with a 3‐mm thickness, were used to produce experimental dumbbell‐shaped specimens (*n* = 10). The specimens were trimmed using a certified manual pressure cutting machine (SOMEH Projects Products and Services), using blades to produce dumbbell specimens according to the ISO 37‐2017 [32], Figure 1A. The specimens produced using DimaPrint (Kulzer, Hanau, Germany) and KeyGuard (Keystone, Gibbstown, USA) were 3D printed using a digital workflow with the same shape and dimensions (*n* = 10) (Table 1). The software (Meshmixer 2017, Autodesk) generated the ISO 37‐2017 stereolithography (STL) file. The STL files were imported into 3D printing preprocessing software (ChiTuBox, V1.9.0, Shenzhen, China). The specimens were positioned at the platform area at a 45° angle, and the 3D printed supports were fabricated (Figure 1B). The printing settings of DimaPrint and KeyGuard specimens were performed according to the manufacturer's recommendations.

**TABLE 1:** Composition of the materials used for mouthguard fabrication provided by manufacturers.

Material	Material type	Composition	Manufacture
Bio‐art	EVA	Ethylene‐vinyl acetate copolymer, Molecular formula (C4H6O_2_·C_2_H_4_) × molecular mass, 114.14, boiling point, < 200°C; melting point (0); 70°C–150°C; Density 0.96 g/cm^3^; CAS registry number 24937‐78‐8	Bio‐art, São Carlos, Brazil
Proform	EVA	Vinyl acetate, CAS 108‐05‐4, EC 203‐545‐4; concentration range 0%–0.3%. Classified as a mixture with no additional identifiers available	Sportsguard Laboratories, Kent, USA
Essence dental	EVA	Thermoplastic flexible ethylene‐vinyl acetate copolymer—no additional information provided	Essence Dental, Ribeirão Preto, Brazil
DimaPrint	3D printed polymer	2‐hydroxyethyl methacrylate: ≥ 25%—≤ 50%; TPO (a photo‐initiator, likely Diphenyl (2,4,6‐trimethylbenzoyl) phosphine oxide): < 3%; Titanium dioxide: < 0.3% (used as a pigment for white resin); Trimethylolpropane triacrylate: < 0.3%	Kulzer, Hanau, Germany
KeyGuard	3D printed polymer	Photopolymer containing methacrylate, photo‐initiator, inhibitor, and pigments. Shore A hardness of 75–90. Tear strength: 30 kN/m, Impact strength: 27.5 j/m^2^. Biocompatible regarding ISO 10993‐5, Cytotoxicity; ISO 10993‐10, Sensitization; and ISO 10993‐23, Irritation	Keystone, Gibbstown, USA

### 2.1 DIMA Print

A layer height of 0.05 mm with a resolution of 100 μm: 55. An ultraviolet‐sensitive (light cured at 405 nm) Dima Print blue polymer (Kulzer, Hanau, Germany) was used on a 3D printer (cara Print 4.0 Pro, Kulzer) to build the models. Before cleaning, a compressed air jet evaporated the residual monomer. The cleaning procedure was performed in isopropanol using the washing machine (cara Print Clean Pro, Kulzer) with pre‐cleaning for 3 min, post‐cleaning for 2 min, drying and post‐curing with cara Print LEDcure (Kulzer, Hanau, Germany) for 5 min on the front and 5 min on the back.

### 2.2 KeyGuard

A layer height of 0.1 mm, bottom layer count of 10, exposure time of 25 s, and bottom exposure time of 60 s. For printing, a 3D printer with 35‐μm XY resolution, LCD monochrome 6.1‐in., 405 nm UV light source, USB connectivity (Sonic Mini 4 K, Phrozen, Taiwan) was used. The cleaning time in isopropanol was 5 min in the Form Wash (Formlabs, Somerville, MA, USA), drying of the samples, and post‐curing process with Form cure (Formlabs) for 30 min at 60°C.

The tensile test for calculating the elastic modulus was performed in a universal testing machine (Eletropulse E3000, Instron, Norwood, USA). The specimen was secured between two pneumatic clamps (2712 Series Pneumatic Action Grips, Instron) (Figure 1C). A non‐destructive axial tensile load from 0 to 150 N was applied with a crosshead speed of 500 mm/min while the load and displacement were recorded in specific software (Blue‐Hill 2, Instron). This load range was selected to ensure that all materials remained within the elastic regime during testing, avoiding any permanent deformation, as previously observed in previous studies [27, 33]. Data from stress–strain curves were collected for each EVA sample and used to calculate the elastic modulus (E, MPa) based on the tangent of the linear portion of the stress–strain curve.

To determine the Poisson's ratio of the tested materials, axial tensile tests were carried out according to ASTM D638 [34]. A constant cross‐head speed of 5 mm/min was applied until approximately 20% strain was achieved. To minimize initial bending effects and ensure proper axial alignment, a 0.1 mm pre‐stretch was applied to each specimen before testing. The stress–strain behavior remained within the linear elastic region during testing, ensuring that Poisson's ratio was determined under small‐strain conditions. To enable accurate measurement of both axial and transverse strain, a random black speckle pattern was sprayed onto the surface of each specimen. High‐resolution photographic images of the region of interest were acquired using a digital macro lens camera (DXM‐l200; Nikon, Japan) (Figure 1D). These images were subsequently analyzed using the open source 2D Digital Image Correlation (DIC) software Ncorr v1.2 (Georgia Institute of Technology, Atlanta, USA) [35].

The density (ρ, g/cm^3^) of each tested material was determined by the standard test method for density and specific gravity of plastics by displacement (ASTM D792) [36]. Each specimen was weighed in air and subsequently immersed in distilled water at 23°C ± 1°C using a precision analytical balance (Marte AY220, Shimadzu, Philippines) (Figure 1E). The procedure involved measuring the mass of the specimen in air, the mass of the immersed specimen, and the mass of the immersion apparatus without the sample.

For mechanical analysis, new dumbbell‐shaped specimens different from those used in the non‐destructive tensile test were used (*n* = 10). The samples were clamped in two grips in a universal testing machine (EMIC DL 3000, Instron) and subjected to a tensile strength test at 50 mm/min speed until rupture (Figure 1F). The elongation (mm) and breaking force (N) were recorded using the TESC‐EMIC software (Instron). The ultimate tensile strength (MPa) was calculated by dividing the rupture force (N) by the specimen cross‐section area (mm^2^). After testing, the failure mode was classified by visual analysis according to the following levels: (I) rupture in the middle area; (II) rupture in the body; (III) rupture in the gripping area.

The Shore A hardness was measured using hardness equipment (Model CV06‐113, CV Instruments, Zervex, Singapore) (Figure 1G), applying a perpendicular force of 10 N for 10 s on five locations of each specimen.

For the scanning electron microscopy (SEM) analysis, the EVA and 3D printed polymer specimens were cut in dimensions of 10 × 10 mm, cleaned in an ultrasonic bath, dried, and gold‐sputtered (QR 150ES, Quorum, Laughton, England). The surface was analyzed using SEM equipment (VEGA 3 LMU, Tescan, Kohoutovice, Czech Republic) with 300× magnification.

**FIGURE 2:**
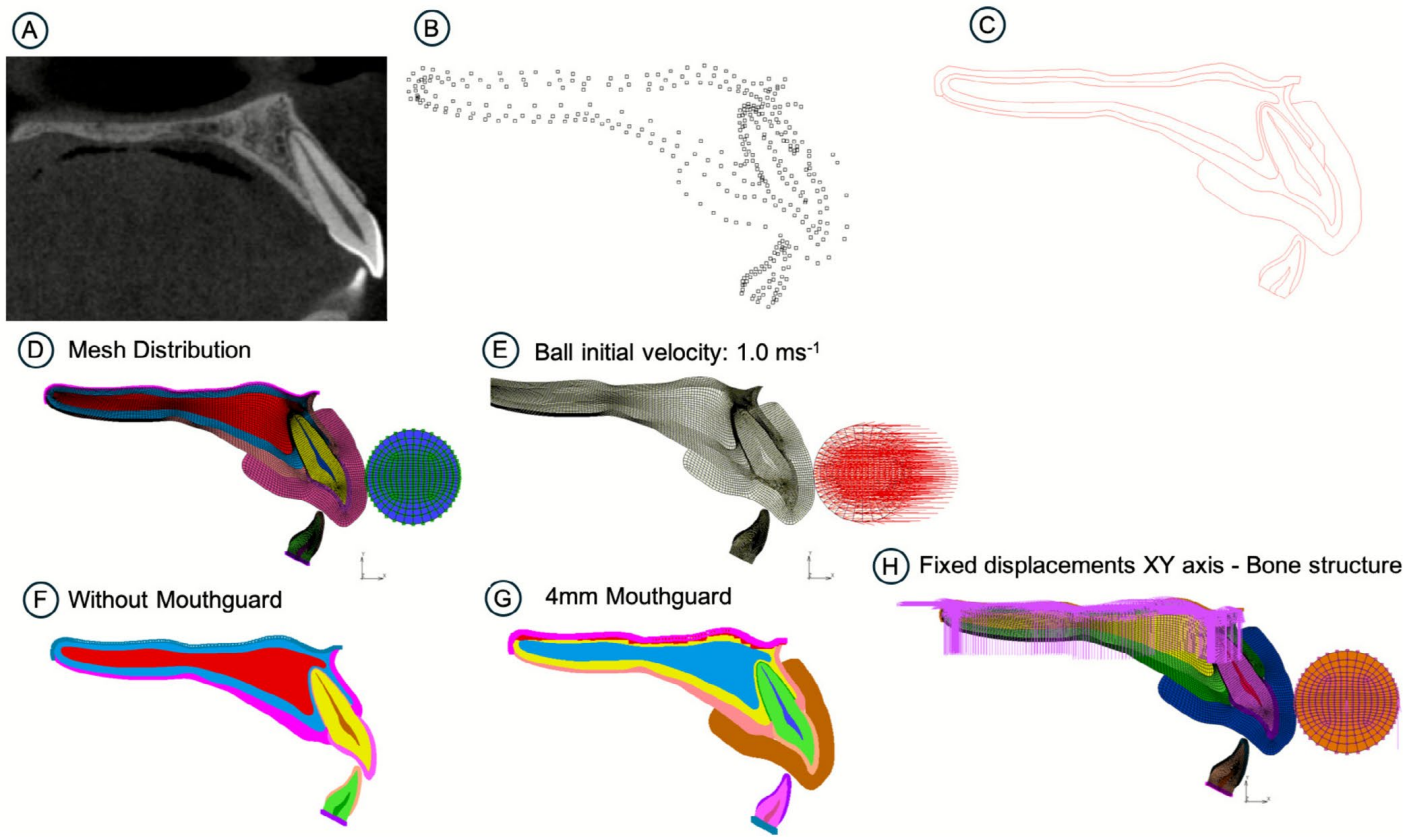
Generation of two‐dimensional finite element models. (A) cone beam tomography image of maxillary central incisor with mouthguard; (B) coordinates points of the CT image imported from Image J; (C) cubic‐spline curves generated from the coordinates; (D) finite element mesh distribution; (E) Ball initial velocity; (F) two‐dimensional model created without mouthguard; (G) two‐dimensional model created with 4 mm mouthguard; (H) boundary conditions.

Two‐dimensional dynamic finite element impact analysis was performed using a model generated by the maxillary central incisor, periodontal ligament, cortical and trabecular bone, soft tissue, and mouthguard (Figure 2). The model was created based on a cone beam tomography image of a patient with normal occlusion (Angle class I, wearing a mouthguard (Figure 2A–C), as described in a previous study [13, 18]. Shock absorption capacity was evaluated at peak strain (maximum impact) for each mouthguard. The shock absorption (%) represents the percentage reduction in peak strain observed in the tooth with a mouthguard compared with the condition without protection, based on the finite element analysis results. It was obtained using the following equation: Shock absorption (%) = ((*ε*_noMG_−*ε*_MG_)/*ε*_noMG_) × 100). Where *ε*_noMG_ is the peak strain value without a mouthguard, and *ε*_MG_ is the corresponding value when the mouthguard is present. In addition, stresses generated in the enamel and dentin were also recorded during impact, following the experimental protocol described [33].

The finite element mesh, composed of four‐node isoparametric quadrilateral plane‐strain elements with reduced integration, was generated using the Marc/Mentat software (Figure 2D). The dynamic impact analysis was performed with the Single‐Step Houbolt method, which is recommended for implicit dynamic contact analyses [18] (Figure 2E). The dynamic impact analysis was performed without (Figure 2F) and with using mouthguard (Figure 2G). The nodes at the base of the bone structure were rigidly constrained in both the x and y directions (Figure 2H). The enamel, dentin, and bone were modeled as orthotropic materials, while the remaining structures were considered isotropic. The mechanical properties (elastic modulus, Poisson's ratio, and density) for dental structure are summarized in Table 2 [37, 38, 39, 40], and for materials used for mouthguard fabrication were calculated experimentally. Frictionless contact was defined between the mouthguard and the tooth surface, allowing separation during impact, while all other interfaces were modeled as bonded.

**TABLE 2:** Mechanical properties applied for dental structures.

Material/structure		Elastic modulus (MPa)	Poisson ratio	Shear moduli	Density (g·cm^−3^)	References
Enamel	Orthotropic	XY plane—63,270 YZ plane—73,720 XZ Plane—63,270	XY plane—0.23 YZ plane—23 0.23 XZ Plane—0.45	20,890	2.14	[18, 37]
Dentin	Orthotropic	XY plane—19,100 YZ plane—19,200 XZ Plane—24,100	XY plane—0.30 YZ plane—0.30 XZ Plane—0.30	8100	2.97	[18, 38]
Periodontal ligament	Isotropic	0.964	0.45		0.95	[18, 39]
Cortical bone	Orthotropic	XY plane—7200 YZ plane—8700 XZ Plane—12,200	XY plane—0.42 YZ plane—0.50 XZ Plane—0.29	2800	2.00	[18, 37]
Cancellous bone	Orthotropic	XY plane—1148 YZ plane—210 XZ Plane—1148	XY plane—0.05 YZ plane—0.01 XZ Plane—0.322	68	0.70	[18, 37]
Soft tissue	Isotropic	10.0	0.30		0.95	[18, 40]
Pulp	Isotropic	2.07	0.45			

The rupture area (mm^2^), maximum force at rupture (N), maximum elongation (mm), ultimate tensile strength (MPa), EVA thickness (mm), and Shore A hardness data were analyzed using the Shapiro–Wilk test to assess normality and the Levene test to verify homogeneity of variances. Then data of each parameter were analyzed using one‐ way analysis of variance (ANOVA) followed by the Tukey post hoc test for pairwise comparisons (*α* = 0.05). All statistical analyses were conducted using a statistical software program (SigmaPlot 12.5; Systat Software Inc). The SEM analysis of the mouthguard specimens was performed qualitatively. The stress (MPa), strain (*με*), and shock absorption (%) capacities of each mouthguard were analyzed descriptively.

## 3 Results

The mean and standard deviation of *F* (N), elongation (mm), UTS (MPa), *E* (MPa), and Shore A hardness for all tested materials are shown in Table 3. One‐way ANOVA showed significant differences among the mouthguards for all measured parameters (*p* < 0.01). Essence Dental had the highest, and DimaPrint had the lowest *F* values (*p* < 0.01). EVA Bio‐art and Proform had similar *F* values (*p* = 0.01), and both had higher *F* values than KeyGuard (*p* < 0.01).

**TABLE 3:** Mean and standard deviation values for EVA plates before thermo‐plasticization measured at non‐contact surface and 3D printed polymers of force (N), elongation (mm), ultimate tensile strength (MPa), elastic modulus (GPa), and Shore A hardness for all tested mouthguard materials.

Material	Material type	Force (N)	Elongation (%)	Ultimate tensile strength (MPa)	Elastic modulus (MPa)	Shore A hardness	Density (g·cm^−3^)	Poison ratio
Bio‐art	EVA	221.2 ± 5.4^C^	325.4 ± 22.1^B^	18.8 ± 1.1^C^	33.7 ± 1.0^B^	82.8 ± 0.3^A^	0.94 ± 0.09^A^	0.29 ± 0.05^A^
Essence dental	EVA	360.7 ± 8.0^A^	316.1 ± 27.2^B^	33.7 ± 0.4^A^	36.8 ± 1.3^B^	81.0 ± 0.6^A^	0.99 ± 0.02^A^	0.29 ± 0.05^A^
Proform	EVA	274.1 ± 8.5^B^	441.8 ± 6.4^A^	23.3 ± 0.6^B^	20.1 ± 0.9^C^	74.9 ± 0.7^B^	0.95 ± 0.01^A^	0.29 ± 0.05^A^
DimaPrint	3D printed polymer	110.1 ± 7.1^E^	24.6 ± 3.8^C^	6.9 ± 0.9^D^	46.5 ± 1.8^A^	74.7 ± 1.1^B^	1.00 ± 0.01^A^	0.39 ± 0.06^B^
KeyGuard	3D printed polymer	136.1 ± 4.8^D^	40.7 ± 3.8^C^	12.9 ± 0.9^D^	45.1 ± 2.3^A^	82.9 ± 0.7^A^	1.02 ± 0.02^A^	0.39 ± 0.06^B^

*Note:* Different uppercase letters mean significant difference among tested materials calculated by Tukey HSD test (*p* < 0.05).

Proform demonstrated the highest elongation, while DimaPrint revealed the lowest values (*p* < 0.01). Bio‐art and Essence Dental exhibited statistically comparable elongation (*p* > 0.05), both of which were significantly greater than that of KeyGuard (*p* < 0.01). Essence Dental exhibited the highest UTS values, while DimaPrint displayed the lowest (*p* < 0.01). Proform exhibited markedly superior UTS values compared to Bio‐art (*p* < 0.01). Bio‐art exhibited superior ultimate tensile strength (UTS) values compared to KeyGuard (*p* < 0.01). DimaPrint and KeyGuard exhibited the greatest E values, whereas Proform demonstrated the lowest (*p* < 0.01). Essence Dental and Bio‐art exhibited comparable E values (*p* > 0.05). KeyGuard, Essence Dental, and Bio‐art exhibited comparable Shore A values (*p* > 0.05) and significantly higher Shore A values than Proform and DimaPrint (*p* < 0.01).

The mean and standard deviation of Poisson's ratio are presented in Table 3. One‐way ANOVA revealed statistically significant differences among the tested materials. The 3D printed polymer group had significantly higher Poisson's ratio values than EVA (*p* = 0.005).

The mean and standard deviation of density values for all materials are presented in Table 3. One‐way ANOVA showed no statistically significant differences among the groups (*p* = 0.055).

The failure mode distribution for the specimens according to the different material types is shown in Table 4. The Chi‐Square test showed significant differences among failure mode distributions (*p* = 0.003). All EVAs had more frequent type II failure. KeyGuard and DimaPrint had predominantly type II and III failures.

**TABLE 4:** Rupture pattern after ultimate tensile strength test for all mouthguard materials tested.

Material	Material type	I	II	III	Chi‐Square ranking
Bio‐art	EVA	0	10	0	A
Essence	EVA	2	8	0	A
Proform	EVA	2	8	0	A
DimaPrint	3D printed polymer	0	5	5	B
KeyGuard	3D printed polymer	0	6	4	B

*Note:* Different letters represent significant differences calculated by Chi‐Square test (*p* = 0.003).

**FIGURE 3:**
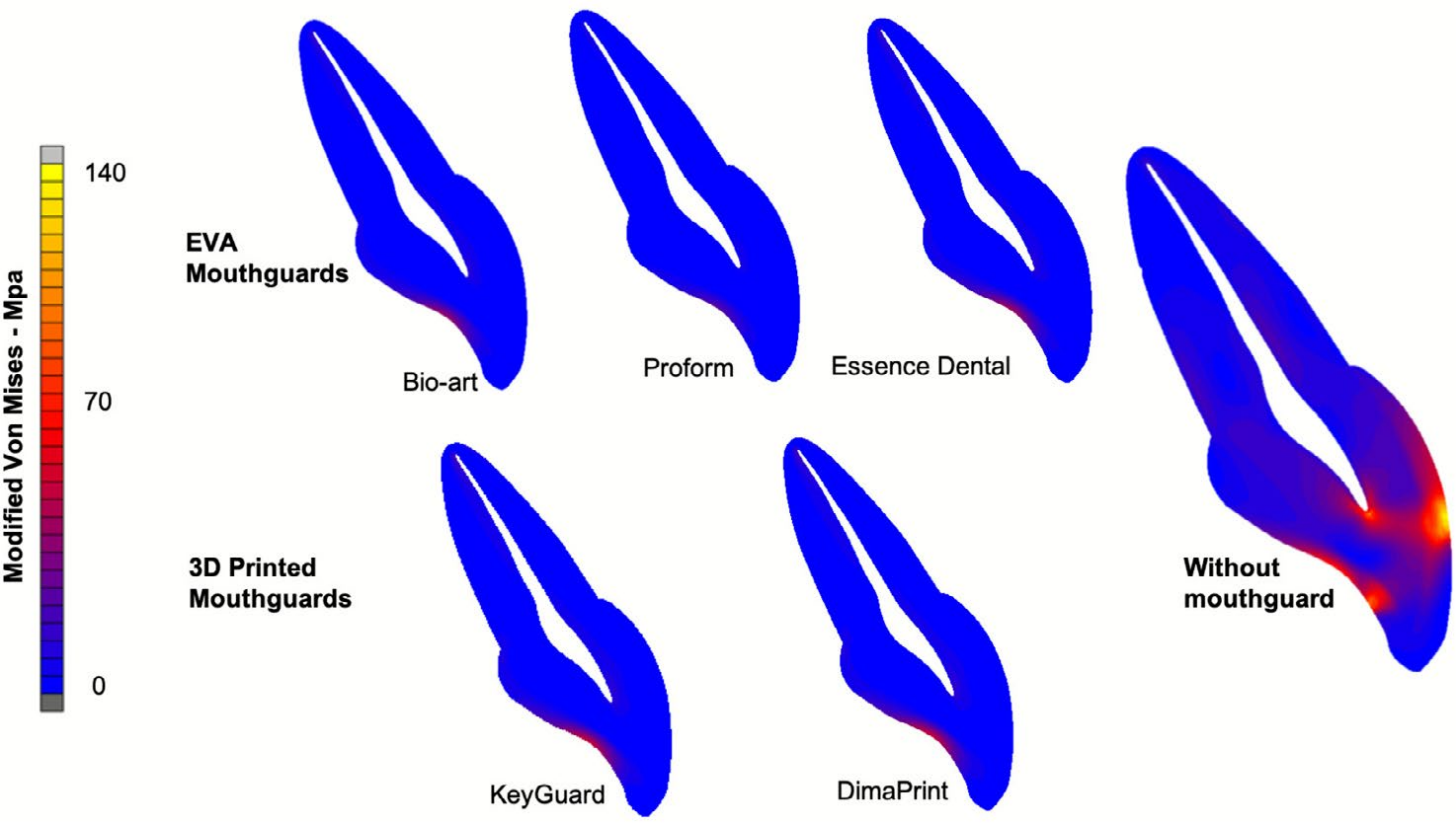
Modified Von Mises stress recorded at the impact peak with 4.0 mm EVA and 3D printed mouthguards and without mouthguard.

The modified von Mises stress distribution values obtained from the finite element analysis are summarized in Figure 3. The model without a mouthguard resulted in the highest stress concentration (140.7 MPa). All mouthguards substantially reduced these values. Among the tested materials, Proform showed the lowest stress value (26.0 MPa), followed by Bio‐art (42.3 MPa), Essence Dental (45.7 MPa), KeyGuard (55.9 MPa), and DimaPrint (56.7 MPa). The mouthguards significantly reduced stress concentration, regardless of type. EVA and 3D‐printed mouthguards showed similar stress reduction.

**FIGURE 4:**
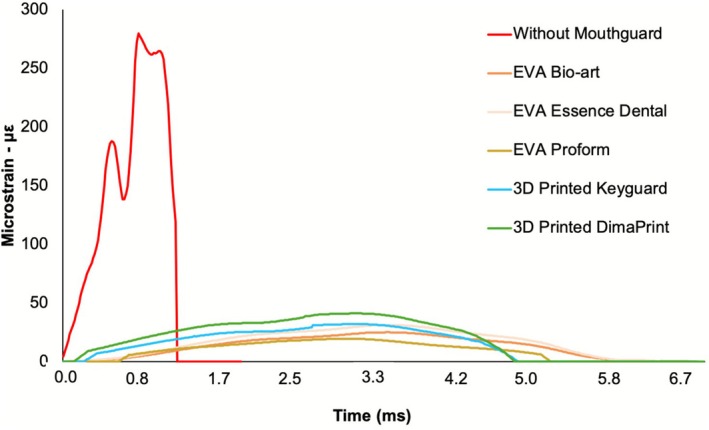
Microstrain values calculated by the finite element analysis during the impact simulation.

**FIGURE 5:**
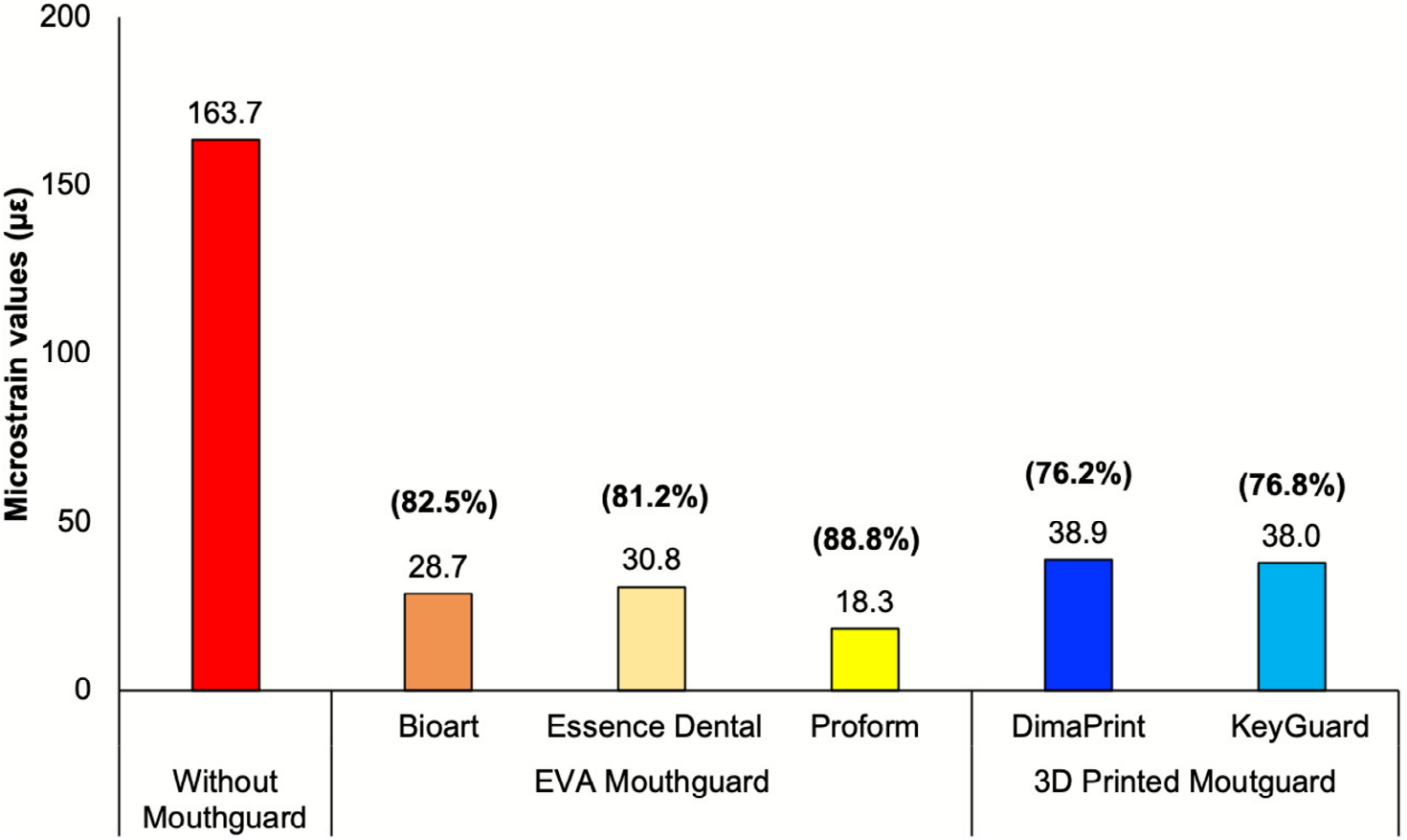
Microstrain values (*με*) and percentage shock absorption in parentheses.

**FIGURE 6:**
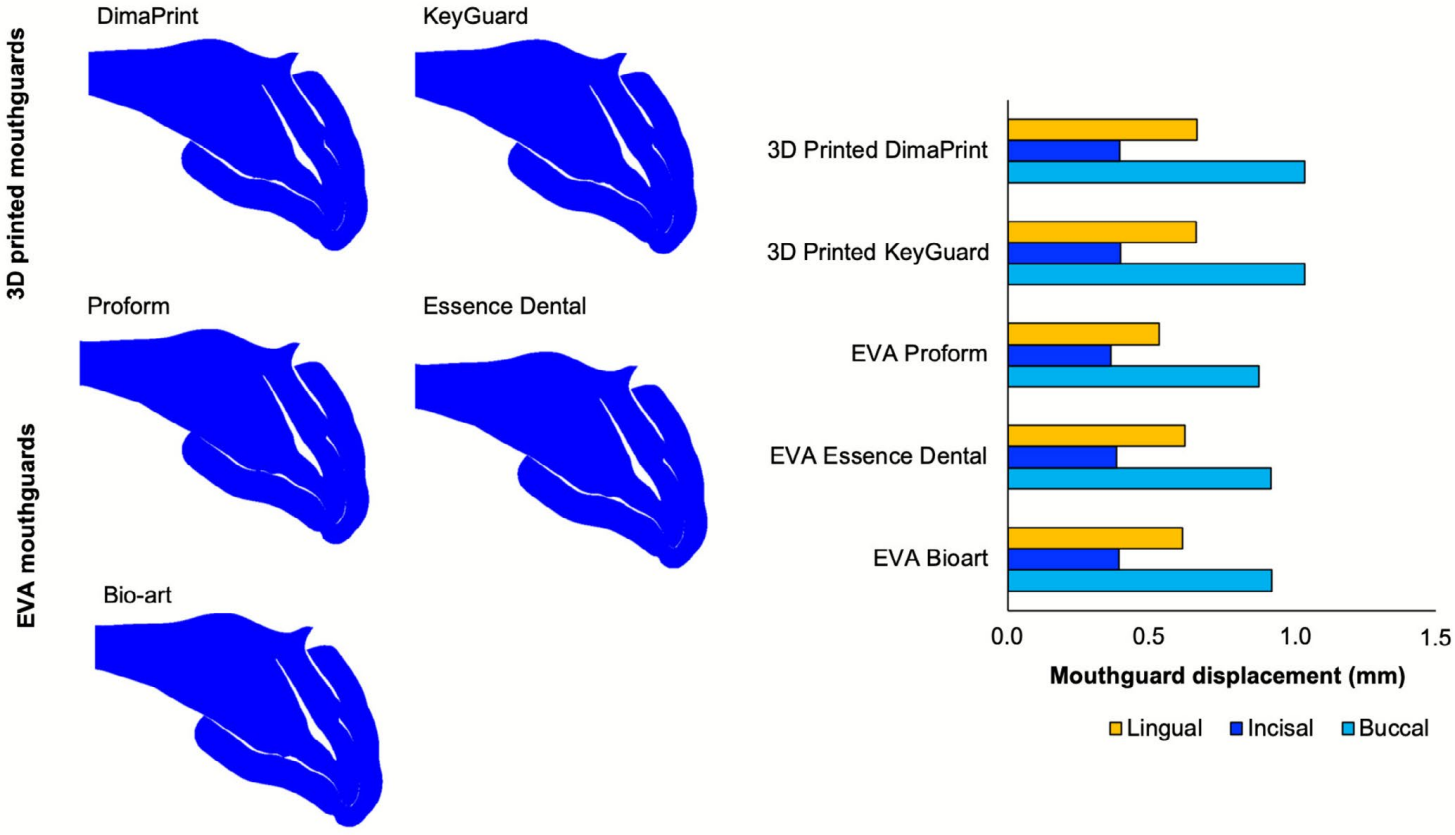
Mouthguard displacement (mm) during the impact application.

The peak strain values and the percentage of shock absorption for each mouthguard type are presented in Figure 4. All mouthguards exhibited similar shock‐absorbing capacity, suggesting comparable impact attenuation. The path plot of mouthguard displacement for each model is shown in Figure 5. Distinct displacement patterns were observed across the buccal, incisal, and palatal regions (Figure 6). The displacement magnitude was minimally influenced by the mouthguard material. Overall, displacement was lower in the buccal region and gradually increased toward the palatal side (Figure 6).

**FIGURE 7:**
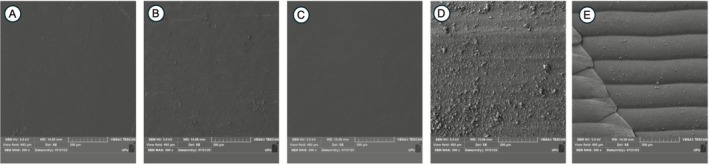
SEM images of the surfaces of EVA and 3D printed polymer mouthguards: (A) Bio‐art, (B) Proform, (C) Essence Dental, (D) DimaPrint, (E) KeyGuard.

Analysis performed using scanning electron microscopy (SEM) revealed notable topographical differences among the evaluated materials. The EVA sheets (Figure 7A–C) exhibited homogeneous surfaces with low roughness and minimal presence of residues or irregularities. In contrast, the 3D printed polymers (Figure 7D,E) displayed markedly rougher topographies. DimaPrint showed the accumulation of surface residues and dispersed granular structures, while KeyGuard presented a stepped pattern characteristic of the 3D printing process, with well‐defined layer lines and embedded particles.

## 4 Discussion

The analysis of the mechanical characteristics and biomechanical performance of 3D printed polymers vs. standard EVA revealed significant differences in the behavior of custom‐made mouthguard materials. The 3D printed polymers DimaPrint and KeyGuard exhibited mechanical behaviors that differed significantly from EVA mouthguards, particularly in terms of maximum force, elongation, and elastic modulus; therefore, the first hypothesis was rejected. However, all tested materials, regardless of their composition, showed a similar ability to reduce stress and strain on the impacted tooth in the finite element simulations, indicating that the second hypothesis was not rejected.

The structural characteristics of each material account for these findings. EVA has a flexible matrix that provides a high capacity for plastic deformation before rupture, thereby enhancing energy absorption [13, 18, 26, 27]. This behavior is reflected in the higher force and elongation values observed experimentally. In contrast, the printed resins develop a highly cross‐linked and predominantly glassy network, which increases stiffness but reduces ductility [41, 42]. As a result, these materials show a greater tendency toward brittle failure and limited elongation, although they exhibit higher surface resistance to indentation.

Significant deviations in Poisson's ratio can adversely impact mechanical performance by reducing the efficiency of stress dissipation [35]. However, despite statistically significant differences observed between groups, variations in Poisson's ratio did not influence the biomechanical effectiveness of the mouthguards in simulated impact scenarios. This finding implies that both EVA and 3D‐printed materials provide comparable protection by effectively minimizing stress transmission to oral tissues.

The alignment of layers inherent to 3D printing, along with the presence of weak interfacial regions between them, may act as stress concentrators and contribute to anisotropic mechanical behavior [43, 44]. The rough and stratified morphology observed in the SEM images of the 3D‐printed mouthguards supports this interpretation, indicating possible weak planes that could affect long‐term mechanical stability and increase susceptibility to fatigue or delamination under cyclic loading. In contrast, the smooth and homogeneous surface of the EVA materials suggests a more uniform structure, which may favor superior durability and stability over time [45]. The results obtained through finite element analysis demonstrated a significant reduction in stress and strain levels in the teeth and adjacent structures when any type of mouthguard was used, regardless of the material. The von Mises stress distributions demonstrated that the absence of a mouthguard led to significant stress concentrations [13, 27, 28], especially in the cervical and buccal regions of the tooth root, areas that are particularly susceptible to fracture following contact [20].

All EVA and 3D printed polymers mouthguards significantly reduced stress, improving load distribution and protecting dental and soft tissues [14, 18, 20, 46]. This finding can be attributed to the fact that, despite exhibiting lower ultimate tensile strength, the 3D printed polymers displayed Shore A hardness values comparable to those of EVAs. This property may have contributed to their ability to protect against deformation under impact, particularly in the regions of direct contact with the dentoalveolar structures [45, 47].

Previous studies have shown that maintaining adequate Shore A hardness can ensure satisfactory impact absorption even in materials with more rigid or brittle mechanical behavior [48]. This helps explain why mouthguards fabricated with 3D printed polymers, despite their inferior performance in other mechanical parameters, were still capable of significantly reducing the stress transmitted to the tooth and surrounding bone during simulated impact [49]. The similarity in Shore A values is a relevant finding, given that this parameter is related to a material's resistance to superficial indentation and its ability to recover its original shape after localized deformation. Furthermore, several studies have emphasized that mouthguard thickness plays a decisive role in impact absorption. A thickness of 4 mm has been widely validated as effective in preventing dental trauma [18, 23, 50]. The use of this standardized thickness for all materials in the present study may explain why, despite their different mechanical properties, similar displacement and stress distribution patterns were observed in the FEA. The 3D‐printed mouthguards represent a promising alternative in contemporary dental treatment, due to their adequate biomechanical performance compared with EVA mouthguards [48, 49]. In contrast to traditional methods that require numerous stages of impression, casting, and thermoforming, often leading to substantial material waste, the digital workflow streamlines the process, markedly diminishing disposable by‐ products like gypsum, surplus plastic sheets, and impression materials [48].

Additionally, the digital model of the mouthguard can be archived and reused, allowing for precise reprinting of the device in the event of loss or wear [48]. This feature represents a significant saving of clinical time and resources and is particularly valuable in high‐risk sports settings, where frequent replacement of protective devices is often required [49]. A limitation of this study is the use of a two‐dimensional finite element model, which, although validated in previous works, does not fully reproduce the three‐dimensional complexity of oral structures. This simplification assumes an extension out of the plane, potentially overestimating overall stiffness and limiting the representation of localized 3D geometrical effects. Moreover, the complete viscoelastic and anisotropic properties of EVA and 3D‐printed polymers were not simulated, which may have led to an underestimation of their capacity for energy absorption and stress dissipation, as viscoelastic behavior plays a key role in impact attenuation. Therefore, the results should be interpreted as comparative trends between materials rather than absolute stress values. Future studies should include complementary in vitro mechanical testing and aging protocols to reproduce the influence of humidity, temperature, and cyclic loading on material degradation, thereby improving the clinical relevance of biomechanical analyses of 3D‐printed mouthguards. Additionally, future investigations should incorporate the viscoelastic properties of the evaluated materials and employ three‐dimensional finite element models to achieve a more accurate representation of the clinical scenario.

## 5 Conclusion

Within the limitations of the study design, the following findings may be drawn. The 3D printed polymers DimaPrint and KeyGuard exhibited distinct mechanical properties when compared with conventional thermoformed EVA materials (Bio‐art, Essence Dental, and Proform). Despite these mechanical differences, all evaluated mouthguards showed a comparable ability to reduce stress and strain on the impacted tooth, confirming their effectiveness in attenuating impact effect under the tested conditions.

## Author Contributions

**Airin Karelys Avendaño Rondon:** experimental development, data acquisition, literature search, data analysis, manuscript preparation and manuscript editing. **Izabela Batista Cordeiro:** experimental development, data acquisition, literature search, manuscript preparation and manuscript editing. **Maribí Isomar Terán Lozada:** experimental development, data acquisition, literature search, data analysis, manuscript preparation and manuscript editing. **Gustavo Mendonça:** conception and design, experimental development, manuscript editing. **Priscilla Barbosa Ferreira Soares:** conception and design, data analysis, manuscript editing. **Paulo Sergio Borella:** experimental development. **Carlos José Soares:** conception and design, literature search, data analysis, funding acquisition, manuscript editing and manuscript revision.

## Funding

This work was supported by Conselho Nacional de Desenvolvimento Científico e Tecnológico, INCT 406840/2022‐9, 422603/2021‐0. Fundação de Amparo à Pesquisa do Estado de Minas Gerais, APQ‐04262‐2, RED‐00204‐23. Coordenação de Aperfeiçoamento de Pessoal de Nível Superior, Finance code 001.

## Conflicts of Interest

The authors declare no conflicts of interest.

## Data Availability

The data that support the findings of this study are available from the corresponding author upon reasonable request.
